# Modified SC Resin Pre-Coating Treatment for Rapid and Robust Repair of CFRP Laminates with Sharp Delamination Cracks

**DOI:** 10.3390/polym17081079

**Published:** 2025-04-16

**Authors:** Yi Chen, Yi Ji, Fei Cheng, Xiaozhi Hu

**Affiliations:** 1 School of Manufacturing Science and Engineering, Southwest University of Science and Technology, Mianyang 621010, China; 2 Department of Mechanical Engineering, University of Western Australia, Perth, WA 6009, Australia; 3 School of Materials Science and Engineering, Southwest University of Science and Technology, Mianyang 621010, China

**Keywords:** CNT reinforcement, edge delaminations, laminated composites, resin pre-coating technique, self-curing resin

## Abstract

A recent composite technique, namely Resin Pre-Coating (RPC), has demonstrated remarkably high effectiveness in the repair of Carbon Fiber-Reinforced Polymer (CFRP) composites. Compared to widely used scarf repair and injection repair, this non-destructive method offers advantages in addressing subsurface damages from the millimeter to micron scale, such as edge delaminations that frequently occur due to machining or low-energy impacts. The acetone-rich RPC solution can spontaneously transport sticky resin and other toughening agents into defects through capillary action. In this study, we further improved the solution by adopting a self-curing resin (i.e., SC-RPC), reducing the repair duration from the initial 2–3 months to merely a few hours. Using this modified solution, the CFRP specimens prepared containing delamination cracks were largely restored, reaching up to 94.9% of the original compressive strength. With the additional incorporation of carbon nanotubes (CNTs), full restoration was achieved, as is evidenced by load-bearing capacities and overload failure modes comparable to those of pristine specimens. The findings of this study may help alleviate concerns regarding substandard post-repair performance and prolonged repair durations, which are frequently criticized in real-world CFRP maintenance projects. The preparation of two new formulations, SC-RPC and SC-RPC+CNT, along with the optimization of key parameters, was carefully detailed in the manuscript to ensure experimental reproducibility.

## 1. Introduction

**Figure 1:**
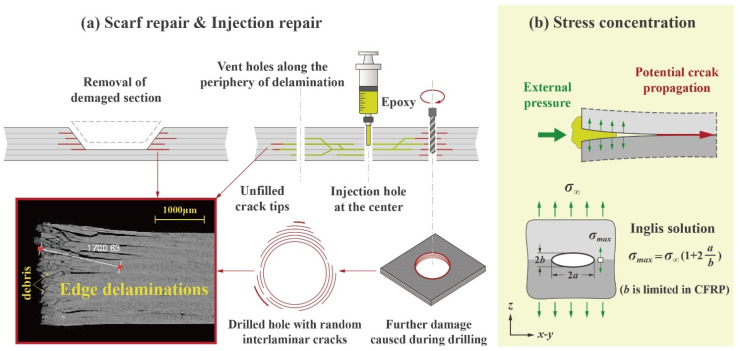
(**a**) Scarf repairs of CFRP require the removal of damaged sections. However, hidden edge delaminations are difficult to fully eliminate, and extra surface grinding may exacerbate them or introduce new ones. (**b**) Injection repairs involving drilling carry similar risks. In addition, due to the poor wettability between carbon fiber and epoxy, external pressure may counterintuitively induce tensile stress, deepening the cracks. The orthotropic multilayer structure of CFRP worsens this situation by confining crack development to the *x*–*y* plane, resulting in an exceedingly large *a*/*b* ratio in the Inglis model [15] and an infinite stress concentration at the sharp crack tip.

Carbon Fiber-Reinforced Polymer (CFRP) laminates have gained increasing utilization across industries including wind energy, automotive and aerospace, owing to their outstanding mechanical properties as well as versatile applicability [1]. While CFRP is now being utilized in some critical components such as engine fan blades, there is a growing concern regarding its reliability and failure behavior [2]. In particular, edge delamination—frequently occurring during cutting, drilling and assembly—has emerged as a major topic of interest, as material engineers have spent decades striving to mitigate this unsolicited damage [3,4]. The schematic in Figure 1a exemplifies how edge delamination cracks can be introduced in CFRP structures, not only in service but also in the maintenance process. These micro-scale interlaminar cracks may not be immediately visible, but they propagate easily under operational loads due to the high stress concentration at the sharp crack tips, creating structural integrity issues and potentially leading to premature failure [5,6].

For repairing damaged CFRP, bolted repair is the current standard for major load-bearing structures, while bonded repairs (e.g., scarf repair and doubler repair) are often preferred when a lightweight or smooth aerodynamic surface needs to be maintained [7,8,9]. Generally, these certified methods are designed to address large-scale delamination or severe damage [10]. Their procedures are complicated and have to be performed by highly qualified personnel with specialized equipment. Even so, small, isolated edge delaminations with very limited crack openings can still be missed if only viscous resins are used. This is because carbon fibers inherently have poor wettability and adsorption with most epoxy systems, stemming from their highly crystallized graphitic basal planes and non-polar surfaces. Also, for this reason, the injection repair widely studied in recent years [11,12,13,14] can hardly be an optimal solution to the problem, as the pressure intended to enhance resin penetrability can instead deepen the cracks, as explained in Figure 1b.

**Figure 2:**
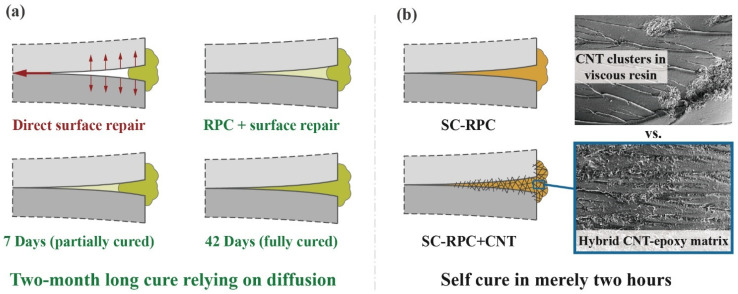
(**a**) While common viscous resins struggle to fill edge delaminations, the RPC solution managed to penetrate and re-bond those narrow cracks; however, it required a month-long period to achieve satisfactory strength through contact curing [16]. (**b**) By using the modified SC-RPC method, the repair duration can be shortened to several hours.

The current study aims to investigate a straightforward, pressure-less repair method known as resin pre-coating (RPC) [16,17,18], which requires no special equipment and can be conveniently performed on-site for both visible defects and small hidden ones. This method was initially developed to improve metal-to-metal or metal-to-composite bonding strength [19] and was later found to be effective also in the repair of composite materials. That is, the acetone-rich RPC solution can penetrate deeply into damaged substrates and leave resin behind at micro-cavities after acetone evaporation. Adopting RPC at the beginning of an adhesive bonding process can facilitate the restoration of mechanical properties by eliminating local stress concentrations, as shown in Figure 2a. For example, early trials on CFRP [20] show that, by simply pre-treating the specimens with an RPC solution consisting of 95 wt% acetone and 5 wt% resin (without hardener), the post-repair compressive strength was further increased by 30% compared to the counterparts with neglected edge delaminations. In addition to CFRP, RPC has also delivered satisfactory results in the repair of other materials, such as granite [21], sandstone [22], glass fiber composites [23] and bamboo composites [24].

Given the proven effectiveness of RPC, we intend to take this technique one step further, preparing it for realistic applications. This requires additional considerations from a practical perspective. For example, early experiments on CFRP [16] revealed a noticeable difference in the repair results at 7 days and 42 days after applying RPC. In reality, even if the two groups yielded equally good results, a week-long curing time is excessive for most maintenance projects. Furthermore, with RPC pre-treatment, the ultimate repair strength achieved after a 42-day curing period did not reach the full strength of pristine specimens [16]. Compared to the economic benefits of attempting repairs instead of direct replacement, the reliability of the repaired structure is often the primary factor to consider.

In response to the above challenges, a self-curing (SC) resin was selected in this study to substitute the conventional resin used in RPC treatment, aiming to establish a rapid and more controlled repair process (referred to as SC-RPC). The possible risks and benefits associated with this resin substitution were examined in the context of the repair of impact-damaged CFRP specimens (with edge delaminations). Besides that, carbon nanotubes (CNTs) were introduced as a supplementary toughening mechanism, as RPC solution also enables the effective dispersion of CNTs, which typically tend to agglomerate in viscous resins [25,26]. By incorporating CNTs, the pure resin matrix was transformed into a CNT–epoxy nanocomposite matrix, which enhances the adhesion with stronger mechanical interlocking at fiber-matrix interfaces, as illustrated in Figure 2b. The optimal SC resin concentration and CNT content, along with their influence on the failure modes of repaired CFRP specimens, were explored in this study.

## 2. Mechanisms for Repair of Sharp Delamination Cracks

### 2.1. Crack/Delamination Propagation Under Internal Stress

Damage in laminated composites often manifests as long, narrow cracks. Therefore, before proceeding with the repair of CFRP, it is necessary to first understand the stress behavior of this type of crack.

Since the proposal of the Kirsch infinite plate problem [27], there has been ongoing interest in characterizing the stress distributions near holes and other discontinuities. For example, in 1913, Inglis developed a simple solution that describes the stress field around an elliptical hole in an infinite plate subjected to remote loading [15], as shown in Figure 1b and Equation (1). Numerically, it might not be as precise as later approaches based on the stress intensity factor. Still, it provides an intuitive understanding of the problem considered in this study.

(1) $$\sigma_{max}=\mathrm{KT}\cdot \sigma_{\infty }=\left(1+\frac{2a}{b}\right)\cdot \sigma_{\infty }$$

where the stress concentration factor KT is used to connect the far-field stress *σ_∞_* and the maximum internal stress *σ_max_* near the ellipse vertex, and KT is approximated as a function of the width (2*a*) and height (2*b*) of the ellipse.

In the context of CFRP laminates, damage initiation and growth in the *x*–*y* plane are promoted by the layered carbon fiber microstructures, while in other directions, they are constrained, i.e., *a* >> *b*. This is particularly evident for edge delaminations arising from sources such as manufacturing defects and low-energy impacts, where *b* → 0. Considering such an extreme case, one can derive an even simpler equation, Equation (2). It clearly indicates that, if these micro-scale interlaminar cracks cannot be effectively sealed through a proper repair process, the infinite stress concentration generated at the unfilled crack tips will easily drive further propagation.

(2) $$\underset{b\to 0}{\lim}\sigma_{max}=\underset{b\to 0}{\lim}\left(\sigma_{\infty }\cdot \frac{2a}{b}\right)=\infty$$

It should be noted that, although Inglis’s linear elastic solution is limited to isotropic materials, the multilayer structure of CFRP further boosts the sensitivity of interlaminar cracks to internal stresses. Therefore, qualitatively, Equations (1) and (2), along with the schematic in Figure 1b, remain relevant for CFRP in illustrating the influence of defects as well as external pressures.

Given this and the poor wettability between carbon fiber and epoxy, it can be inferred that the widely studied epoxy injection may not be the best choice for repairing CFRP in many cases, as even weak injection forces can introduce internal stresses within edge delaminations, triggering infinite stress concentrations at unrepaired crack tips. This underscores the advantages of pressure-less repair methods, such as RPC.

### 2.2. Repair and Reinforcement Mechanisms of RPC and RPC+CNT Treatments

The standard adhesive system (a mixture of epoxy and hardener), commonly used in the manufacturing and repair of CFRP and other fiber composites, is characterized by its high viscosity. As a result, it struggles to seal micro-defects like edge delaminations. Instead, the recommended RPC solutions (e.g., 5 wt% resin plus 95 wt% acetone) can adequately penetrate the cracks and deliver ideal repair results, as reported in [18]. This is because acetone, as a strong organic solvent, possesses great flowability and can carry epoxy resin without altering its properties.

In this study, to improve the curing efficiency of RPC solutions, an SC resin has been adopted to replace the conventional resin previously used. The newly formulated SC-PRC solutions contain up to 70–95 wt% acetone, with the remaining components consisting of the SC resin and CNTs as a supplementary enhancement phase. In principle, these acetone-rich solutions can spontaneously transport sticky resin and CNTs into narrow cracks, relying on capillary action rather than external pressure. However, due to the poor wettability between carbon fiber and epoxy system, sufficient capillary pressure is essential to fill not only the sharp crack tips but also the secondary voids left behind by the escaped acetone.

Capillary action is often characterized by the height to which the liquid climbs within a thin tube. Alternatively, the capillary height *h* can be determined based on surface tension *σ_s_* and contact angle cos*θ* when it comes to the interaction between the liquid and a specific solid, e.g., RPC solution and CFRP. The relationship between these parameters has been derived by Young and Laplace [28,29], as outlined below.

(3) $$\Delta P=\rho gh=\frac{2\sigma_{s}\cos\theta }{R}\to h=\frac{2\sigma_{s}\cos\theta }{R\rho g}$$

The capillary pressure, symbolized by Δ*P*, acts as the driving force of fluid transport. It is related to the hydrostatic pressure within the tube, exerted by the liquid column of diameter *R*, height *h* and density *ρ* [30].

**Figure 3:**
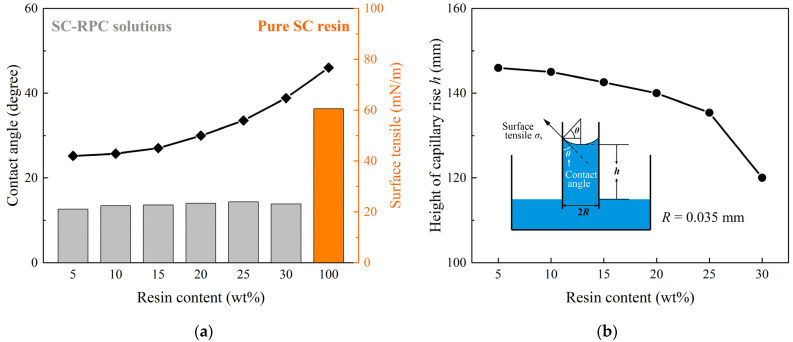
(**a**) Measurements of contact angle and surface tension between SC-RPC solutions and CFRP surfaces, performed using an optical contact angle goniometer (OCA 100, Dataphysics, Germany). (**b**) Estimates of capillary rise height *h* of SC-RPC solutions with varying resin content.

Preliminary experiments showed that mixing acetone into the SC resin significantly improved the wettability and flowability of the solution, reducing the surface tension by half or more as illustrated in Figure 3. In turn, the viscosity of the solution recovered as we raised the resin content, and it was observed that the downward trend in capillary height *h* became more pronounced when the resin content was raised from 25 wt% to 30 wt%. This could be attributed to the accelerated increase in the contact angle between SC-RPC solutions and CFRP surfaces. So, before further evaluating the performance of SC-RPC solutions at various concentrations, a resin content range of 5 to 25 wt% appears to be a reasonable interval that aligns with the mechanism underlying this method.

Additionally, this acetone-rich solution offers the advantage of enhancing CNT dispersion, which can be achieved through a few minutes of mechanical stirring. As shown in Figure 2b, CNTs (typically nanosized in diameter and microscale in length), along with the adhesive resin, are drawn into delaminated cracks via capillary action and are well-distributed within the crack micro-architectures. The CNTs that enter the substrate can further improve joint performance by transforming the pure epoxy matrix into a CNT–epoxy nanocomposite matrix and re-establishing mechanical interlocking in the z-direction (between adjacent fracture surfaces). Such an effect is usually difficult to achieve, which lays the theoretical foundation for elevating repair strength to a new level.

### 2.3. Benefits and Potential Issues of Replacing Conventional Resin with SC Resin in the RPC Repair Process

**Figure 4:**
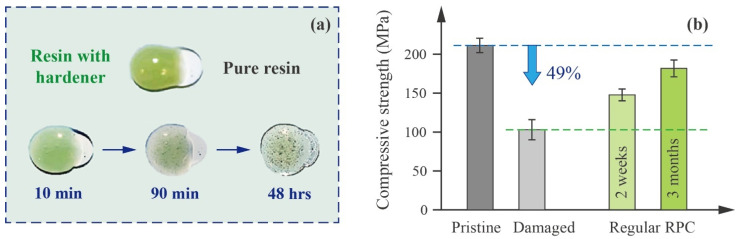
(**a**) Contact diffusion of resin–hardener mix to pure resin (reproduced with permission from Ref. [18]), which is also the mechanism behind well-studied self-healing composites [32]. (**b**) However, the curing performance relying on such contact curing is unstable, particularly due to its strong dependence on time, making it less suitable for routine repair applications.

As shown in Figure 4a, in the conventional RPC process, the resin deposited in defects solidifies through a “contact curing mechanism”. That is, the hardener migrates from a high-concentration region (the resin–hardener phase subsequently applied on the surface) to a region of low concentration (the pre-deposited resin phase) until equilibrium is reached throughout the space. According to Han’s research [18], contact curing is effective but inefficient, as its performance depends on multiple factors, including temperature, pressure and, most importantly, duration. The study [18] reports that achieving a moderate level of strength requires at least two weeks, while attaining optimal repair strength takes up to three months, as given in Figure 4b. An additional issue overlooked in the literature is that micro-cavities with varying geometry and surface roughness can cause anisotropic diffusion of the hardener, which further leads to uneven resin curing and potential phase segregation. This might be a key factor contributing to the failure of early RPC trials [16].

In principle, the issues mentioned above can be avoided by using the new SC resin. This resin substitution can significantly reduce the curing time, possibly achieving satisfactory strength within hours. Such a change greatly enhances the practical value of the RPC technique from an engineering perspective. Although the modified SC-RPC solution requires certain heating conditions to initiate the curing reaction, it can be satisfied through common portable infrared heating lamps, as SC-RPC is intended primarily for small-scale structures or larger structures with localized damage. Overall, SC-RPC is easy to operate and highly flexible, with minimal requirements for equipment and personnel. It can be carried out either independently or in combination with other repair methods (e.g., scarf repair), depending on the size, shape and function of the damaged structure.

However, several concerns remain to be addressed in the present study. First, while the SC resin helps reduce the repair duration, it is necessary to double check the potential adverse effects of acetone dilution on its curing properties [31]. Second, the post-repair strength achieved using SC-RPC needs to be assessed. Does it match the strength of pristine specimens, as raised in the Introduction? Lastly, the adjustment of key parameters, such as the optimal resin concentration and the number of treatment cycles, requires clarification.

## 3. Specimen Design and Experimental Methods

### 3.1. Preparation of CFRP Specimens with Edge Delamination Cracks

The CFRP panels used in this study were commercially sourced from Carbonwizer Technology Co., Ltd. (Shenzhen, China). They are made of 11 layers of T300 carbon fiber prepregs, including 9 unidirectional layers oriented at 0°/90° as well as 2 twill weave layers positioned at the top and bottom. During preparation, these panels were trimmed into 67 mm × 42 mm pieces. After being impacted at the center, each piece was further bisected into two strips, with extensive delamination cracks concentrated along the cutting boundary.

**Figure 5:**
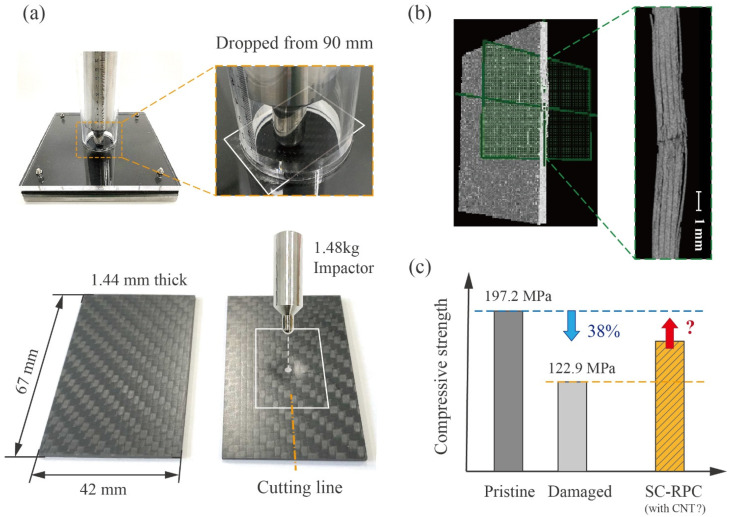
(**a**) The drop hammer apparatus was used to induce impact damage at the center of the CFRP panel, which was then bisected into two symmetrical CFRP strips with delamination cracks along the cutting boundary. (**b**) CT scans confirmed the presence of extensive subsurface microcracks, several millimeters long and a few microns wide. (**c**) The damaged CFRP strips retained only approximately 60% of their original compressive strength. To what extent can they be restored using the proposed SC-RPC solution?

A custom-designed tube drop hammer impact tester was used to induce delamination cracks in the CFRP specimens prepared for repair, as depicted in Figure 5a. It delivered an impact energy of approximately 13 J through a 1.48 kg hammer released from a height of 90 cm. A 3 mm-thick rubber cushion was placed beneath the specimen to control energy dissipation. The impact indentation is barely visible on the front and back surfaces; however, CT images in Figure 5b revealed a significant proliferation of delamination cracks, accompanied by fiber ruptures, concentrated around the impact center. These sharp cracks extended up to 8 mm in length, while their width ranged from a few to tens of microns. Compressive testing, conducted in accordance with ASTM D3410/D3410M-03 [33], indicated that edge delamination cracks caused a 38% reduction in the strength of the CFRP strips, as illustrated in Figure 5c.

### 3.2. Preparation and Application of SC-RPC Solution

The SC-RPC solution is a simple mixture of SC resin, CNTs (if required) and acetone. The SC resin, which serves as the primary restorative agent, is formulated with a bisphenol A diglycidyl ether-type epoxy resin and a latent hardener (a modified dicyandiamide). The CNTs, designed to construct crack-interface bridges, are short multi-walled CNTs with a nanoscale diameter of 30–50 nm and a microscale length of 1–10 μm, supplied by Nanjing XFNANO Materials Tech Co., Ltd. (Nanjing, China). Finally, fast-evaporating acetone, with a purity exceeding 99.5%, acts as the carrier.

To begin, the damaged CFRP specimens were immersed in the SC-RPC solution for 15 s, allowing the acetone-diluted SC resin to penetrate deep into the delamination cracks via capillary action, as explained in Section 2. The soaked specimens were then heated in an oven at 70 °C for approximately 20 min to evaporate the acetone. Typically, three to four cycles were performed to ensure thorough filling of the sharp crack tips and the voids left by the escaped acetone. Since the SC resin cures more easily and rapidly than conventional resins, the heating temperature and duration have been carefully adjusted to prevent premature solidification between cycles, thereby avoiding the formation of unwanted boundaries within the CFRP.

Following the above pre-treatment process, a standard surface repair of the delaminated edge was performed by applying a layer of pure SC resin. Finally, the coated specimens were pre-cured at 130 °C for 1 h, followed by post-curing at 170 °C for 0.5 h. Light polishing and ultrasonic cleaning were conducted in between to remove any excess resin adhering to the side faces of the repaired specimens.

A range of resin concentrations (5–25 wt%) was tested to determine the optimal formulation, based on which different CNT contents (0.2–1.6 wt%) were tried to maximize the repair strength of the CFRP specimens. The wettability of the newly formulated SC-RPC solutions with varying parameters was examined, as well as the potential detrimental effects of acetone dilution on the curing properties of the SC resin. Appendix A detailing specimen grouping has been provided at the end of the paper.

### 3.3. Examination of CFRP Specimens with or Without Repair

To evaluate the effectiveness of the SC-RPC solution in repairing delamination cracks, the in-plane compressive properties of the CFRP specimens, with and without repair, were examined using a Changchun Kexin WDW100 universal testing machine (Changchun, China). Following ASTM D3410/D 3410M-03 [33], the strip specimens were clamped by two tensile fixtures 25 cm apart, with the repair area (or damaged area) positioned at the center. The loading rate was set to −1 mm/min, and the load–displacement curve was recorded every 0.1 s. The results are included in Appendix A.

Additionally, the CFRP specimens, before and after repair, were scanned using an X-ray micro-computed tomography system (FF35 CT, YXLON, Hamburg, Germany) at 195 kV tube voltage and 110 μA tube current. A 3D delamination profile was generated by rotating the specimen 360°, i.e., the detector captures numerous 2D projected images from which a 3D dataset of volume elements was reconstructed based on algorithms. Visualization and an analysis of the data were performed using VGSTUDIO MAX 3.1 software.

## 4. Results and Discussion

### 4.1. Influence of Acetone Dilution on the Curing Properties of SC Resin

Although acetone-diluted resin solutions have shown promising results in certain substrate bonding and repair applications [22], the introduction of a new resin matrix in this study necessitates additional investigation into the potential adverse effects of acetone on its curing properties. For example, Loos [34] observed that residual acetone can compromise the stiffness of epoxy resin by interfering with the cross-linking process. Similarly, Mondragon [35] reported that even trace amounts of resin diluent (e.g., CH_2_Cl_2_) can affect the mechanical performance and glass transition temperature by altering the epoxy network structure. To address these concerns, FTIR analysis (PerkinElmer Spectrum One, USA) was conducted to examine the molecular structures of SC resins before and after RPC treatment. Such analysis ensures that the proposed SC-RPC method can be confidently accepted in composite repair for stronger adhesive bonding.

**Figure 6:**
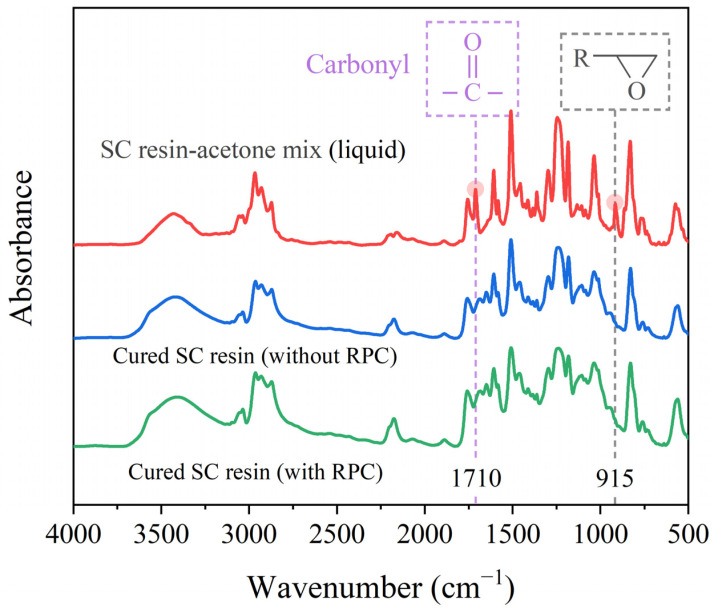
Infrared spectra of the SC resin–acetone mix (in liquid) and the cured SC resins with and without RPC treatment. The carbonyl group indicates the presence of acetone in the resin solution or specimen, while the epoxy group at 915 cm^−1^ reflects the curing state of the resins.

The infrared spectra of the SC resin–acetone mixture (liquid) and the cured SC resins, both with and without RPC treatment, are compared in Figure 6. This figure illustrates the infrared rays absorbed by different molecular groups, where the x-axis is the wavenumber indicating the location of the absorption peak and the y-axis is the absorbance reflecting the absorption intensity. For example, the carbonyl group, characterized by a peak between 1680 cm^−1^ and 1750 cm^−1^, is a key functional group in acetone [36]. This group is absent in the as-received SC resin, and its presence would indicate residual acetone within the tested samples. As shown in Figure 6, the carbonyl sketching was observed exclusively in the SC resin–acetone mixture, confirming that, in the designed RPC process, acetone completely evaporated before the resin hardened. In fact, such observation served as an indicator during the optimization of the heating temperature and duration, as discussed in Section 3.2. Another key finding from Figure 6 is that the infrared spectra of the two cured SC resins are nearly identical in terms of band locations and peak intensities. Judging from previous experience [31], the adhesive joints formed with as-received and RPC-treated SC resins are expected to achieve comparable mechanical performance. The CFRP repair results presented later in this study tend to support this hypothesis.

**Figure 7:**
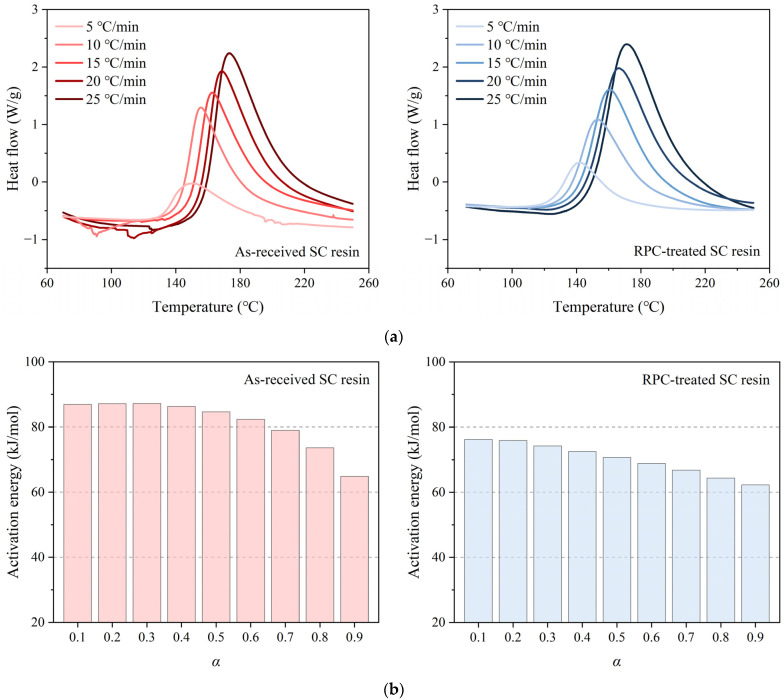
(**a**) The DSC curves of as-received and RPC-treated SC resins, along with (**b**) their activation energies calculated using the Kissinger method [37,38].

Since both resins, treated and untreated, require a heated environment to initiate curing, their temperature-related properties were further analyzed using differential scanning calorimetry (DSC, TA Q200, USA), as shown in Figure 7. The results indicate that the two resins exhibited similar heat transfer patterns, but they slightly differed in the activation energy required at various curing stages (denoted by *α*). For the regular SC resin, the activation energy [37,38] remained high during the initial stages and began to decrease once *α* reached approximately 0.5. This turning point likely corresponded to the opening of epoxy rings, which released a significant amount of hydroxyl groups that, in turn, accelerated the curing reaction. In contrast, repeated tests consistently showed that the RPC-treated SC resin required lower activation energy throughout the reaction period, displaying a steady downward trend from the outset. This suggests that the modified resin has the potential to achieve thorough curing in a shorter time. It is inferred that the addition of acetone to the viscous SC resin prevented the slow separation of epoxy resin and curing agent (dicyandiamide) due to density differences, thereby enhancing their contact and promoting the formation of a robust epoxy network. This unexpected finding is valuable and aligns with the objectives of this study.

### 4.2. Effectiveness of SC-RPC Solutions in the Restoration of Compressive Strength of CFRP Specimens with Sharp Delamination Cracks

This modified SC-RPC method, which replaces conventional resin with SC resin, eliminates the need for contact curing and significantly reduces curing time. As a result, the success of repairs now hinges primarily on two factors: the resin concentration in the SC-RPC solutions and the number of treatment cycles applied. This shift allows for a more controlled and predictable restoration of CFRP strength.

In this section, SC-RPC solutions with varying resin concentrations (5–25 wt%) were evaluated for repairing CFRP specimens. Building on previous works [16], initial repair attempts employed a three-cycle SC-RPC process to ensure adequate resin penetration and defect filling as acetone evaporated. This was followed by a refinement phase, where the number of treatment cycles was adjusted based on the optimal resin concentration identified.

**Figure 8:**
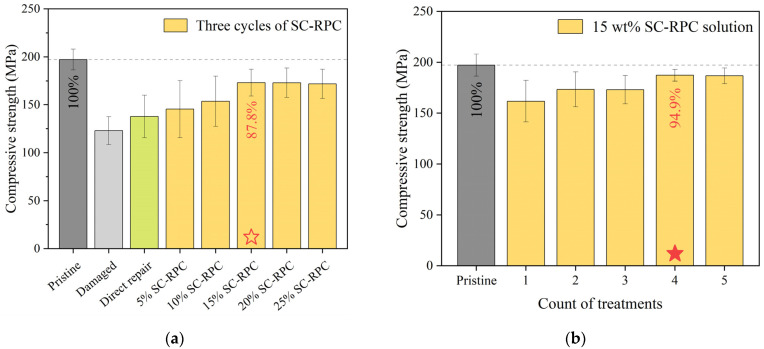
(**a**) As expected, adopting SC-RPC at the beginning of the standard surface repair process facilitated the restoration of compressive strength in CRRP specimens. In addition, by comparing the repair effects of the SC-RPC solutions, the optimal resin concentration was identified. (**b**) Using the optimized 15 wt% SC-RPC solution, the repair strength increased steadily over four treatment cycles but tended to stabilize in the fifth cycle.

Figure 8a presents the compressive strengths of specimens pre-treated with different SC-RPC solutions, alongside reference values for pristine specimens, damaged specimens and directly repaired specimens. The results indicate that after impact, the compressive strength of CFRP specimens dropped to approximately 60%. Specimens that underwent direct surface repair (epoxy with hardener) regained 70% of their original strength. In contrast, specimens pre-treated with SC-RPC solutions, even at low concentrations, were able to achieve an additional 5–10% strength recovery, demonstrating that SC-RPC is indeed beneficial for sealing narrow and elongated defects easily formed in carbon fiber composites. Upon increasing the resin concentration to 15 wt%, the repair strength reached 88% of the original—nearly three times the effectiveness of direct surface repair. In addition, three consecutive cycles of 15 wt% SC-RPC treatment largely restored the integrity of CFRP specimens, as evidenced by reduced variation in strength values.

However, it is worth noting that further increases in resin concentration yielded no more improvement, with compressive strength plateauing between 87% and 89%. One possible explanation for such a phenomenon is that excessively high resin concentrations hindered acetone evaporation, compromising adhesive joint quality. Another plausible reason is that even after three cycles of SC-RPC treatment, the deposited resin was insufficient to fully fill and consolidate delamination cracks. The test data in Figure 8b appear to support the latter hypothesis: when employing a 15 wt% SC-RPC solution, CFRP specimens exhibited a consistent enhancement in compressive strength through four treatment cycles, followed by performance stabilization during the fifth cycle. This series of tests helped us determine the key parameters suitable for our case, which can serve as a reference for establishing parameters for other carbon fiber composites produced using different designs and manufacturing processes.

**Figure 9:**
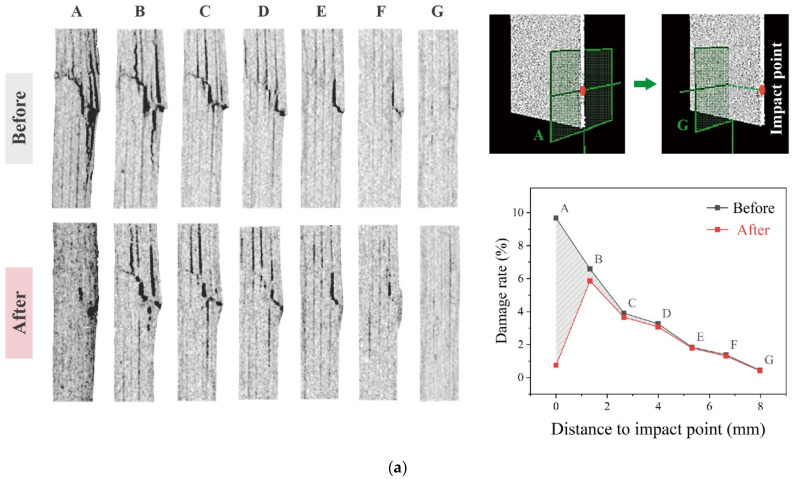

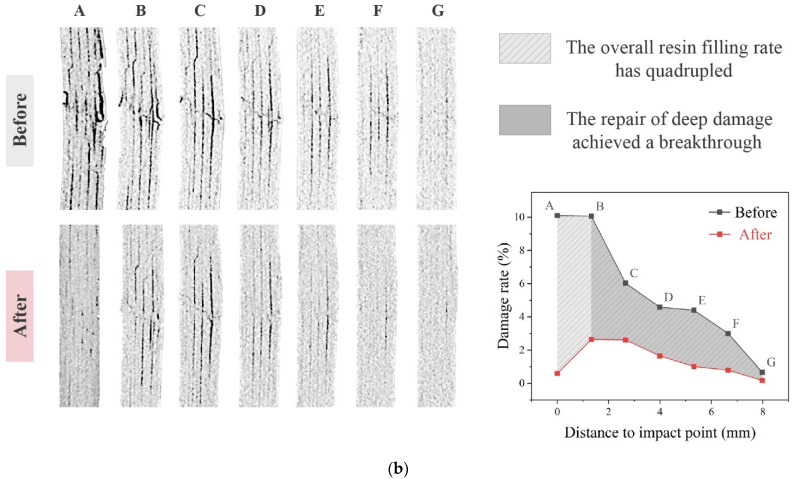
SC scanning results of the CFRP specimens repaired with and without SC-RPC treatment. Only by completely filling the defects and resolving the stress concentration problem can the strength and reliability of CFRP specimens/structures be substantially restored. (**a**) Direct surface repair (resin with hardener). (**b**) Repair with one SC-RPC treatment cycle.

CT scanning results given in Figure 9 further elucidate the strength changes associated with SC-RPC treatment. The pre- and post-repair crack morphologies observed across various sections of the CFRP specimens—from the impact surface (Position A) to a deep section near the delamination front (Position G)—provide insights into the repair mechanism and effectiveness of different approaches.

Despite variations in crack patterns between the two CFRP specimens in Figure 9, comparative images clearly show that the specimen pre-treated with SC-RPC received a more comprehensive repair. Particularly, images from sections D, E and F (4–7 mm from the surface) show that the crack shapes in Figure 9a remained almost identical before and after repair, while the post-repair cracks in Figure 9b became visibly shorter and narrower. This highlights the unique capability of SC-RPC in addressing deep damages in carbon fiber composites. The evolution of the delamination front, as detected by CT scanning, aligns well with the previously described SC-RPC mechanism, i.e., acetone-rich SC-RPC solutions spontaneously transport sticky resin into narrow crack tips via capillary action.

Furthermore, digital image processing and statistical analysis revealed that, following a single SC-RPC cycle, the resin filling rate in impact-induced defects increased by 300%, especially achieving a breakthrough in the coverage of deep damages. However, it is important to interpret these results carefully, as the corresponding strength change was not as pronounced (see Figure 8). This, once again, underscores the importance of adequate penetration and consolidation of delamination cracks; otherwise, even a high filling rate does not fundamentally resolve the stress concentration issue that persists in CFRP specimens. For example, as in Figure 8, the groups with low resin concentrations, as well as those with insufficient SC-RPC treatment cycles, all ended up with limited enhancement in strength. Moreover, their strength values displayed notable scatters, even greater than that observed in the un-repaired group. Therefore, the SC-RPC method presented in this study encompassed not only a conceptual understanding but also a series of meticulously designed and tested procedural steps.

### 4.3. CNT Reinforcement Within Sharp Delamination Cracks Deposited Through SC-RPC Treatment, Referred to as SC-RPC+CNT

As an additional toughening mechanism, CNT reinforcement was introduced into delamination cracks through SC-RPC treatment. Compared to flexible fibrous structures, these straight and rigid nanotubes are more effective at bridging narrow carbon fiber gaps (both intrinsic and impact-induced gaps). When combined with the epoxy system, they cure into a CNT hybrid nanocomposite, wherein CNTs intertwine with carbon fibers to form a complex and robust network.

**Figure 10:**
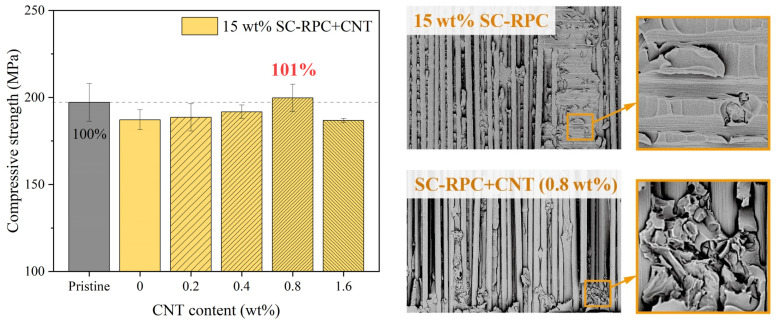
Together with the SC-RPC treatment, an appropriate amount of CNT doping further enhanced the in-plane compressive strength of the repaired CFRP specimens. Meanwhile, SEM observations revealed that the fracture surfaces of CNT-toughened joints were noticeably rougher compared to that of pure resin joints.

Through strength comparisons and observations of CNT-toughened adhesive joints (see Figure 10), SC-RPC+CNT has been confirmed as an optimal method for addressing sharp delamination cracks within CFRP. The experimental data show that incorporating CNT networks further improved the compressive strength by 2–12 MPa. Notably, when the CNT content was increased to 0.8 wt%, the average strength of the repaired specimens matched that of the pristine specimens—achieving 100% restoration. Furthermore, new cracks formed during compressive loading did not overlap with the impact dents, with some even initiating outside the repair area. The fracture surfaces became significantly rougher, indicating increased energy absorption during failure. All these details sum up what we anticipated: the repair strength exceeded the original strength, and the observed strength increment (2–12 MPa) does not fully capture the potential of the SC-RPC+CNT method.

Nonetheless, the CNT content in the SC-RPC solution must be carefully controlled. Excessive doping (e.g., 1.6 wt%) can result in aggregation within carbon fiber gaps, hindering the complete filling of irregular and narrow cracks. Beyond CNT content, the shape, size and strength of CNTs can also influence the reinforcement outcomes, which was not explored in this research but will be evaluated in future studies to understand their repair effects on CFRP damage.

**Figure 11:**
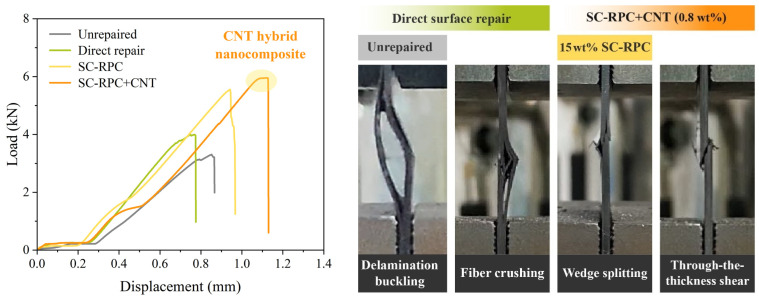
Compressive load–displacement curves of damaged and repaired CFRP specimens under compression, together with their typical failure modes.

In addition to the improvement in strength values, SC-RPC treatment as well as CNT doping also led to immediate changes in the failure modes of CFRP specimens. Figure 11 lists the typical compressive stress responses and failure modes of specimens prepared in four separate conditions. In this final section, a brief fractography analysis is conducted to review the mechanisms behind each repair method.

According to the test results, CFRP specimens subjected to compression-after-impact predominantly failed by delamination buckling. It often initiated the growth of delamination at the outermost layers (on the impact side), as the impact weakened the local matrix stiffness and compromised the lateral stability of these layers. Sub-laminates then formed within the ligament, acting as new bearing elements. They are typically slender structures and are susceptible to buckling failure. Alternatively, bending moments due to off-center loading can also cause transverse fractures, as shown in Figure 11.

Direct surface repair had a limited effect on the compressive strength restoration of impacted CFRP specimens. Instead, the main change was observed in the failure mode, which shifted from global buckling to localized fiber crushing. The epoxy resin coated onto damaged surfaces recovered lateral support to the outer layers, which to some extent helped inhibit or delay widespread buckling. However, numerous impact-induced microcracks remained within the specimens. As pressure continued to increase, these random cracks propagated and interconnected, quickly dividing the laminate into multiple independent sub-laminates. Consequently, after the linear elastic phase, fiber crushing progressively occurred at various panels near the impact site, leading to premature failures with highly scattered peak loads.

Wedge splitting and through-the-thickness shear are failure modes commonly observed in high-performance CFRP manufactured using autoclave-cured prepregs. In principle, if the resin matrix and fiber/matrix interfaces are effectively repaired and reinforced in the prepared specimens, uniform stress distribution will be re-established in them and these failure modes will reappear. In this test, wedge splitting was exclusively observed in specimens pre-treated with SC-RPC. It is characterized by two distinct shear fractures propagating through the thickness, forming a wedge and a delamination where they meet. Since shear fractures cannot extend beyond delamination, it is speculated that delamination occurred first due to weakened interlaminar strength. In contrast, through-the-thickness shear, which corresponds to the maximum load-bearing capacity, prevailed in specimens repaired using the SC-RPC+CNT method, highlighting the superiority of hybrid joints formed by interlaced carbon nanotubes and carbon fibers. Therefore, it is foreseeable that the combination of SC-RPC solution and CNT reinforcement holds important practical value for repairing damage in CFRP and other fiber-reinforced composites.

## 5. Conclusions

Compared to scarf repair and injection repair commonly used for severe damages in CFRP structures, the non-destructive RPC technique is tailored for sealing minor and deep defects, such as edge delaminations. It possesses inherent advantages in addressing stress concentration issues, which can be crucial in projects focused on structural reliability and post-repair lifespan. The primary task of this study involves resin substitution. The new SC resin eliminates the reliance on contact curing, enabling a rapid cure within hours while maintaining equal curing performance. This makes the modified SC-RPC a competitive option for CFRP maintenance, capable of being applied independently or in combination with other methods to achieve optimal repair outcomes. The key findings of this study are summarized as follows.

(1) According to FTIR observations, acetone dilution did not pose detrimental effects on the new SC resin in terms of the molecular structure and curing properties. Conversely, it enhanced the thermal performance of the resin, smoothing the DSC curves and reducing the activation energy required for the curing reaction.
(2) As the primary control group, direct surface repair did prevent widespread buckling of the CFRP specimens. However, it fell short of addressing the subsurface defects and the associated stress concentration issues, resulting in a limited restoration of compressive strength (about 10%). Even low-concentration SC-RPC solutions brought further improvements, as they managed to penetrate delamination cracks (up to 8 mm in length, but only a few microns in width).
(3) Through a series of comparative experiments, it was observed that after four cycles of 15 wt% SC-RPC treatment, the compressive strength of the CFRP specimens reached a peak point, approximately 95% of the original strength. The repair rate was tripled compared to the control group, with a significant breakthrough in the coverage of deep damages, as revealed by CT images.
(4) A 100% restoration was achieved by incorporating a tiny amount of CNTs (0.8 wt%) into the SC-RPC solution. Hybrid adhesive joints, with interlaced carbon nanotubes and carbon fibers, strengthened mechanical interlocking at the interfaces. As uniform stress transmission was re-established, typical failure modes, e.g., wedge splitting and through-the-thickness shear, reappeared on the repaired CFRP specimens. Note that our example did not fully reflect the potential of SC-RPC+CNT, as the fresh fractures initiated away from the repair regions.

The preparation of SC-RPC solutions and SC-RPC+CNT suspensions, as well as the optimization of the resin/acetone ratio and CNT content, have been meticulously detailed, which may serve as a reference for further long-term reliability tests or the repair of other composite materials.

## Data Availability

The experimental data have been included in Appendix A.

## Appendix A

See Table A1 for the experimental data.

**Table A1:** Repair conditions and results of various CFRP specimen groups.

#	Group Description	Count of Specimens	Count of SC-RPC Treatment Cycles	Compressive Strength (MPa)	
				Average	Standard Deviation
1	Pristine	10	NA	197.16	10.89
2	Impacted	10	NA	122.98	14.53
3	Direct repair	7	NA	137.82	22.09
4	5 wt% SC-RPC	8	3	145.52	29.70
5	10 wt% SC-RPC	8	3	153.55	26.15
6	15 wt% SC-RPC	8	3	173.05	13.91
7	20 wt% SC-RPC	8	3	173.00	15.28
8	25 wt% SC-RPC	8	3	171.81	15.19
9	15 wt% SC-RPC	8	1	161.73	20.47
10	15 wt% SC-RPC	8	2	173.42	17.08
11	15 wt% SC-RPC	8	4	187.19	5.69
12	15 wt% SC-RPC	8	5	186.72	7.60
13	15 wt% SC-RPC +0.2 wt% CNT	8	4	188.57	7.84
14	15 wt% SC-RPC +0.4 wt% CNT	7	4	191.77	3.89
15	15 wt% SC-RPC +0.8 wt% CNT	8	4	199.71	7.89
16	15 wt% SC-RPC +1.6 wt% CNT	8	4	186.83	1.09
